# Real-time assessment of triage nurse situational awareness (SA) using the situation awareness global assessment technique (SAGAT)

**DOI:** 10.1371/journal.pone.0318555

**Published:** 2025-02-12

**Authors:** Abeer Abdulaziz Alfuraydi, Modi Al-Moteri

**Affiliations:** 1 Medical Surgical Nursing Department, College of Nursing, Taif University, Taif, Saudi Arabia,; 2 Emergency Department, Main Hospital, King Salman Bin Abdulaziz Medical City, Madinah Region, Saudi Arabia; Istanbul University - Cerrahpasa, TÜRKIYE

## Abstract

**Background:**

The emergency department (ED) is a complex, unpredictable, and distraction-filled environment. Within these challenging conditions, triage nurses are tasked with quickly assessing, identifying, and prioritizing patients who require urgent interventions. Studies from other safety-critical fields, such as aviation and the military, have highlighted that situational awareness (SA) is a critical skill for optimizing human performance during time-sensitive situations. In these fields, limitations in SA have been identified as significant risks to safety. However, despite its importance, SA in the context of EDs has been largely overlooked. Endsley’s SA theory and the Situation Awareness Global Assessment Technique (SAGAT) provide a framework to evaluate SA in dynamic and complex workspaces. This study aims to assess SA during real-time triage processes of patients in EDs, utilizing Endsley’s SA model.

**Method:**

An observational cross-sectional study was conducted to assess SA. Forty real-time triaging processes were observed and evaluated in two ED sites, using the SAGAT to measure SA levels.

**Results:**

A total of 40 triage nurses participated in the study across two ED sites. The findings revealed that the perception of patient cues was significantly reduced by workload (p = 0.048) and stress (p = 0.025), while playing video games was associated with enhanced perception of patient cues (p = 0.014). Additionally, a significant negative correlation was observed between the perception of patient cues and comprehension, indicating a cognitive trade-off between these two SA levels. Only 10% (n = 4) of participants achieved a good SA score, emphasizing the need for improvement.

**Conclusion:**

The results underscore the importance of understanding and improving situational awareness in triage nurses using Endsley’s SA model. These findings offer valuable insights for enhancing future practice, education, and research focused on optimizing situational awareness in emergency nursing.

## Introduction

The emergency department (ED) environment is inherently complex, unpredictable, and characterized by frequent distractions [1]. Within these challenging conditions, triage nurses must rapidly assess, interpret, and prioritize patients requiring urgent medical intervention [2]. The effectiveness of triage processes is critically dependent on nurses’ ability to continuously monitor and synthesize relevant information concerning the patient’s condition, including any sudden or unexpected changes [3].

Situational awareness (SA) is defined as “the perception of the elements in the environment within a volume of time and space, the comprehension of their meaning, and the projection of their status in the near future” [4,5]. Studies across various safety-critical domains, such as aviation, road safety, and the military, have demonstrated that SA is a vital skill for understanding and optimizing human performance in time-sensitive and high-stakes scenarios [6–8]. Similarly, the healthcare industry has adopted the concept of SA to describe the cognitive processes that underpin efficient and safe decision-making in complex, dynamic, and high-risk environments [9]. Endsley’s SA model provides a comprehensive framework for assessing SA in dynamic and complex work settings. This model delineates SA into three hierarchical levels:

1. **Level 1**: Perception of the environment, which involves gathering information from multiple sources, including patient-related data, electronic health records, and input from other clinicians.
2. **Level 2**: Comprehension of the meaning of information and events, requiring the synthesis, interpretation, and prioritization of data to develop an accurate understanding of the current system state.
3. **Level 3**: Projection of future states, involving the anticipation of potential outcomes and trajectories, enabling contingency planning for high-probability scenarios.

By systematically addressing these levels, Endsley’s SA model offers valuable insights into evaluating and enhancing the situational awareness of individuals in complex work environments.

In clinical practice, SA is integral to ensuring patient safety, as it forms the foundation for decision-making, clinical judgment, reasoning, and performance [10,11]. SA enables nurses to identify and report abnormal clinical cues in a timely manner, make accurate and prompt decisions, and respond effectively to patient deterioration incidents [11]. This capability is particularly critical in emergency practice, where immediate and accurate triage decisions are essential [12,13].

However, maintaining SA poses significant challenges, as it can be easily compromised in situations where nurses are busy, fatigued, bored, overly excited, or anticipating future events [3–5]. The loss of SA often results in the failure to recognize and communicate clinical cues promptly, thereby increasing the likelihood of medical errors [14,15]. Research has identified the loss or reduction of SA as a leading cause of real-time errors, frequently culminating in poor performance and adverse patient outcomes [3]. Conversely, maintaining high levels of SA has been shown to mitigate such risks and improve patient outcomes [11]. Given its importance, nurses working in EDs must possess a robust sense of SA. Operationalizing SA within the ED context is essential to ensuring the delivery of efficient and safe care [16,17].

SA and triage decision-making are shaped by a combination of clinicians’ intrinsic limitations (e.g., working memory, mental models, and alertness) [16], situational constraints (e.g., complexity and uncertainty), and the dynamic interplay between these factors [11]. SA failures, which are relatively common, pose significant risks to patient safety, underscoring the importance of implementing strategies to enhance nurses’ ability to maintain high levels of SA. Despite its critical role, SA remains a largely overlooked aspect of workplace safety in EDs [16].

Currently, there is limited evidence on the prevalence and incidence of SA failures in EDs, as well as on the internal and external factors contributing to these failures. This gap in understanding hampers the development and implementation of effective interventions to address SA reduction. In their study, Al-Moteri et al. [3] emphasized the need for further research focused on the human limitations of SA within nursing practice. In alignment with this recommendation, the present study seeks to assess SA during real-time triage processes in EDs, utilizing Endsley’s SA model as the theoretical framework.

## Method

A cross-sectional study was conducted to assess SA by observing real-time triage processes in the EDs of two Ministry of Health (MOH) tertiary hospitals. The study spanned a three-month period, from December 18, 2022, to February 20, 2023. Participation in the study was entirely voluntary, with all participants providing informed consent prior to their involvement.

### Participants and settings

All triage nurses employed in the emergency departments of Hospital A (n = 80) and Hospital B (n = 100) were deemed eligible to participate, resulting in a total eligible population of 180 nurses. Invitations to participate were extended to all eligible individuals, and 40 triage nurses consented to participate in the study.

The ED of these two Ministry of Health (MOH) tertiary hospitals have a combined capacity of 86 beds, with 41 beds in Hospital A and 45 beds in Hospital B. On average, Hospital A manages approximately 320 emergency patient visits per day, while Hospital B handles around 300 visits. During peak periods, these numbers increase to approximately 400 visits per day in Hospital A and 350 in Hospital B.

### Data collection tools

Two types of data collection tools were utilized for this study: (1) an 8-item demographic questionnaire designed to collect information on age group, sex, work experience, frequency of playing video games, prior training, shift timing, mental stress, and workload; and (2) the Situational Awareness Global Assessment Technique (SAGAT) [4,5]. The following sections provide detailed explanations of these data collection instruments.

#### Demographic questionnaire.

The demographic questionnaire collected information on participants’ age, sex, and years of work experience in the emergency department, which was used to create an experience variable. The frequency of playing video games was also recorded, as prior research suggests that such games may enhance attentional skills [18]. Alsaad et al. [18] and other studies in cognitive sciences [19] and healthcare education [20] indicate that video games, especially those requiring rapid decision-making and visual tracking, can improve visual search abilities and attention to detail. These abilities are directly relevant to situational awareness (SA), particularly in the fast-paced and high-pressure environment of emergency departments.

Additional variables were incorporated based on evidence from the literature. These included whether participants had undergone life support training, such as Basic Life Support (BLS), Advanced Cardiovascular Life Support (ACLS), or Pediatric Advanced Life Support (PALS), as well as whether they had experienced mental stress resulting from interruptions or workload associated with shift overcrowding. Life support training has been demonstrated to enhance situational awareness (SA) by equipping individuals with essential knowledge and skills. Conversely, mental stress [21] and excessive workload [22] are well-documented factors that can negatively affect attention focus, thereby potentially compromising SA.

#### SAGAT.

SA was measured using the SAGAT [4,5], which is well-documented in the literature for its validity [4,5]. SAGAT assesses an individual’s three levels of SA—perception (Level 1 SA), comprehension (Level 2 SA), and projection (Level 3 SA). When constructing SA items for emergency nurses during the triage process, the requirements were defined as the dynamic information needed to accomplish the triage task, rather than fixed information like policies and guidelines. Hogan et al. [23] developed the SAGAT with three items at Level 1, one item at Level 2, and three items at Level 3. Additionally, Gardner and colleagues [24] used three items for each level of SA at each freeze to assess the SA of medical trainees.

In this study, the SA items were formulated according to Endsley’s process [4]. A pool of 35 queries targeting the three levels of SA was initially constructed. The items were piloted before the study to ensure representativeness and feasibility. Five questions were removed from the SAGAT items due to inapplicability. The remaining items were reviewed and assessed by three subject matter experts (SMEs), including one in the SA area and two ED nurses with five years of experience. The SMEs rated most queries (27 items) as relevant, yielding a CVI of 0.87. Three items received CVIs less than 0.6 and were removed. The final 27 items, as shown in Table 1, included three items at Level 1, two items at Level 2, and one item at Level 3 in each freeze.

**Table 1:** Sociodemographic characteristics of the emergency nurses (n = 40).

Study variables	N (%)
Age group	
20–30 years	19 (47.5%)
31–40 years	16 (40.0%)
41–50 years	05 (12.5%)
Sex	
Male	05 (12.5%)
Female	35 (87.5%)
Emergency work experience	
0–1 year	05 (12.5%)
2–5 years	31 (77.5%)
6–10 years	02 (05.0%)
>10 years	02 (05.0%)
Life support training	
BLS	40 (100%)
Advanced life support training^†^	
ACLS	13 (32.5%)
PALS	05 (12.5%)
Video games	
Never	12 (30.0%)
Rarely	07 (17.5%)
Sometimes	17 (42.5%)
Always	04 (10.0%)
Shift time (8-hour shift)	
Morning (7am to 3pm)	09 (22.5%)
Afternoon (3pm to 11pm)	11 (27.5%)
Night (11pm to 7am)	20 (50.0%)
Physical workload	
Somewhat load	02 (05.0%)
Moderately load	13 (32.5%)
Very load	12 (30.0%)
Extremely load	13 (32.5%)
Mental stress	
Somewhat stressful	09 (22.5%)
Moderately stressful	09 (22.5%)
Very stressful	10 (25.0%)
Extremely stressful	12 (30.0%)

**Fig 1:**
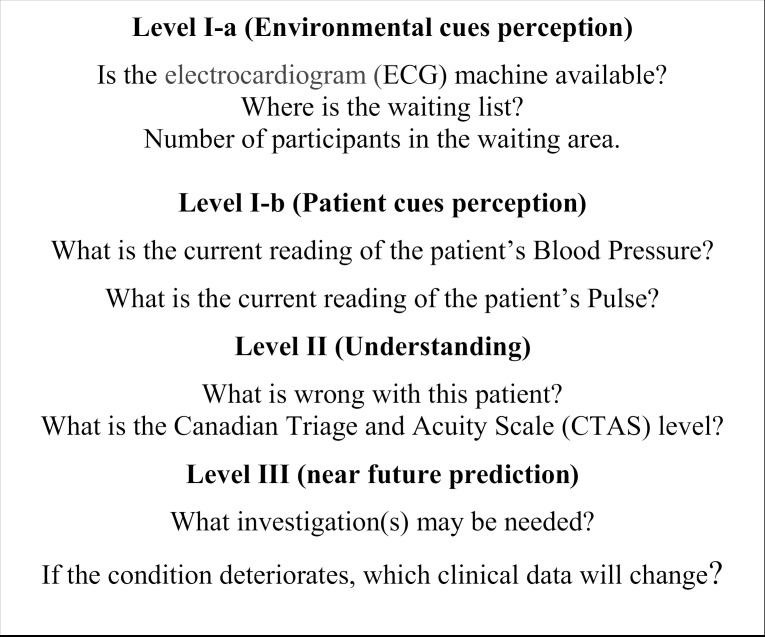
Situation awareness SAGAT queries.

To assess a nurse’s comprehension level, the “Canadian Triage and Acuity Scale (CTAS)” level was included as one of the items. The CTAS is a widely used triage tool in emergency rooms in Saudi Arabia to prioritize patient care based on the urgency of their condition. Its integration into this study ensures alignment with standard triage practices in the region, making the results directly relevant to local ED workflows. The SA queries were designed to be broad and customizable for emerging situations, such as asking about patient cues (“What is the current reading of patient …?”) or environmental cues (“Where is the … located?”). The environmental cues were primarily linked to prior manipulation of the triage room equipment by the observer. Fig 1 provides examples of the SAGAT queries. Confidence in attending to situational cues was rated on a 5-point Likert scale ranging from “very confident” to “not at all confident.”.

### Data collection process

All eligible participants were approached via institutional email. The email addresses included the study aim and brief explanatory statement. Only those who showed interest in participating in the study were consented. It is worth noting that no prior assessment of participants’ conceptual understanding or baseline knowledge of situational awareness (SA) was conducted before their inclusion in the study. The primary focus of this research was to evaluate participants’ SA during real-time triage processes, emphasizing their natural responses and workflows within the emergency department. This approach was intended to preserve the authenticity of the observed SA levels while avoiding the introduction of additional educational or evaluative biases prior to data collection. Since all participants were to be observed by the primary researcher of the current study, a written time-table plan for data collection was designed based on actual emergency department work staffing and scheduling.

A “freeze probe technique” [4,5] was employed to observe participants in the triage room during real-time triage processes. This method was carefully integrated into the natural workflow of the ED to minimize disruption while maintaining observational validity. The SAGAT was administered during these “freezes” by randomly presenting pop-up questions to participants. The questions were specifically designed to assess participants’ situational awareness without altering their typical task execution patterns.

To align the freeze probe method with the ED’s dynamic workflow, freezes were timed to occur during non-critical moments in the participants’ tasks. This timing ensured that the freezes did not interfere with patient care. The pauses lasted approximately 30 seconds each and were introduced in a manner that simulated real-world interruptions commonly experienced in ED settings, such as sudden requests for information or guidance.

During a freeze, participants were asked task-relevant questions designed to measure their perception, comprehension, and projection of situational elements. Examples of these questions include: “Where is the ECG machine located?”, “What is the current blood pressure reading of the patient?”, and “Which patient clinical findings pose a threat to the patient’s clinical condition?”. The integration of these questions within the participants’ workflow was validated by ensuring the scenarios and queries mirrored typical ED operations, thereby supporting the observational validity of the method.

Additionally, before administering the SAGAT, the workflow and freeze process were pilot-tested to confirm representativeness and feasibility. Feedback from emergency department stakeholders ensured that the process did not impede the usual triage workflow. By using this rigorous approach, the freeze probe technique provided an authentic assessment of situational awareness in a real-time clinical environment while preserving the validity of the observational data collected.

To ensure consistency, participants’ responses were double-checked for confirmation during data collection. No patient-identifiable information was collected throughout the process. Demographic data were obtained following each SAGAT administration. Perception and comprehension levels were calculated by comparing participants’ responses during the observation with information recorded by the researcher for each item, categorized as correct or incorrect. Projection levels were verified in collaboration with the ED physician assigned to the patient. The total SA score was derived as the aggregate of the perception, comprehension, and projection scores.

### Ethical approval

Permission to conduct the study was obtained from the Scientific Research Ethics Committee (IRB22-076), which oversees all research activities within the administrative cluster, including both hospitals involved in the study. Both hospitals operate under a single administrative and ethical governance structure, thereby eliminating the need for separate IRB/REC approvals for each site. Additionally, the heads of the EDs at both hospitals were contacted to discuss the study protocol and ensure its alignment with their respective workflows. During these discussions, permissions were shown, and the application of the study procedure was clarified. Written informed consent was obtained from each participant before his/her participation in the study. Written informed consent from each participant before his/her participation in the current study was collected. Additionally, participants were reassured that the study was non-evaluative and aimed at understanding situational awareness rather than assessing individual performance.

## Analysis

All statistical analyses were conducted using the Statistical Package for Social Sciences (SPSS) version 26 (Armonk, NY: IBM Corp., USA). Quantitative variables were expressed as means and standard deviations. The study’s independent variables consisted of participants’ sociodemographic characteristics, while the dependent variables were their SA levels. Relationships between the independent and dependent variables were assessed using Fisher’s exact test. Comparisons between the SA scores and sociodemographic characteristics, as well as nurses’ experiences in the emergency department, were performed using the Mann–Whitney Z test and the Kruskal–Wallis H test. Statistical collinearity was evaluated using both the Shapiro–Wilk and Kolmogorov–Smirnov tests. The SA scores exhibited a nonnormal distribution; therefore, nonparametric tests were applied. Additionally, Spearman’s correlation was conducted to explore relationships between global perception, patient information perception, comprehension, and projection. Statistical significance was defined as p < 0.05.

## Results

### Demographic data

Table 1 presents a descriptive analysis of the participants’ demographic data and their perceptions of the triage room work conditions during the time of data collection. Among the 40 participants who consented to participate, 87.5% (n = 35) were female, and 47.5% (n = 19) were between the ages of 20 and 30 years. Additionally, 77.5% (n = 31) reported having two to five years of work experience in the emergency department. All participants held Basic Life Support (BLS) certifications, while 32.5% (n = 13) and 12.5% (n = 5) had also completed ACLS and PALS training, respectively.

Regarding video game usage, 42.5% (n = 17) of the participants reported that they sometimes or always played video games. Concerning work conditions, 32.5% (n = 13) and 30.0% (n = 12) perceived their shift at the time of data collection as very or extremely loaded, respectively. Additionally, 25.0% (n = 10) and 30.0% (n = 12) described the moment of data collection as extremely stressful. A detailed breakdown of the demographic data is provided in Table 1.

### SA score

Table 2 presents a descriptive analysis of the participants’ SA. Among the 40 participants, only 10% (n = 4) achieved a good SA score. Specifically, 52.5% (n = 21) of participants correctly perceived environmental cues, while 50.0% (n = 20) accurately perceived patient-related information. Additionally, 62.5% (n = 25) were able to comprehend and relate this information effectively, and 67.5% (n = 27) utilized the cues to project future scenarios. Regarding confidence levels, approximately 50.0% (n = 20) of participants expressed confidence in attending to situational cues.

**Table 2:** Assessment of Situational Awareness Global Assessment Technique (SAGAT) (n = 40).

Variables	Correct N (%)
Level 1-a: Environmental perception	21 (52.5%)
Level 1-b: Patient information perception	20 (50.0%)
Level 2: Comprehension	25 (62.5%)
Level 3: Projection	27 (67.5%)
Total awareness score (mean ± SD)	2.33 ± 0.92
Level of awareness	
Poor (score 0–1)	06 (15.0%)
Acceptable (score 2–3)	30 (75.0%)
Good (score 4)	04 (10.0%)
Confidence level	
Somewhat confident	05 (12.5%)
Moderately confident	15 (37.5%)
Very confident	11 (27.5%)
Extremely confident	09 (22.5%)

### The relationship between different variables

Table 3 illustrates the relationships between participants’ perceptions of environmental cues, patient cues, comprehension, projection, and their sociodemographic characteristics. The analysis revealed a statistically significant relationship between participants’ perception of patient cues and playing attention games (p = 0.014), workload (p = 0.048), and stress levels (p = 0.025). No significant associations were found between environmental cue perception, comprehension, or projection and the sociodemographic characteristics.

**Table 3:** Association between environmental and patient information perception and the sociodemographic characteristics and experiences of the nurses in the ED department (n = 40).

Factor	SA levels			
	Environmental perception	Patient information perception	Comprehension	Projection
Age group	0.342	0.527	0.745	1.000
Sex	0.172	1.000	0.633	0.307
Emergency work experience	0.607	0.605	1.000	0.584
Video games/week	1.000	0.014**	0.477	0.716
Shift time	0.918	1.000	1.000	1.000
Physical workload	0.745	0.048**	0.746	1.000
Mental stress	0.525	0.025**	0.747	0.185
Confidence level	1.000	0.752	1.000	1.000
ACLS training course	0.738	1.000	0.730	0.157
PALS training course	1.000	1.000	0.633	1.000

§ P value was calculated using Fisher’s exact test.

** Significant at the p < 0.05 level.

Table 4 investigates the total SA score in relation to the sociodemographic characteristics and experiences of the participants. The analysis did not reveal any statistically significant relationships between the overall SA score and factors such as age group, gender, work experience, playing attention games, shift timing, physical workload, or mental stress. A detailed breakdown of these associations is presented in Tables 3 and 4.

**Table 4:** Association between SA score and the sociodemographic characteristics and experiences of the nurses in the ED department (n = 40).

Factor	Awareness Score (4) Mean ± SD	Z test	P value^§^
Age group			
20 – 30 years	2.26 ± 0.65	0.707	0.728
>30 years	2.38 ± 1.12		
Sex			
Male	2.00 ± 1.41	0.348	0.402
Female	2.37 ± 0.84		
Emergency work experience			
≤5 years	2.33 ± 0.83	0.866	0.879
>5 years	2.25 ± 1.71		
Attention games			
Yes	2.39 ± 0.92	0.346	0.760
No	2.17 ± 0.94		
Shift time			
Morning	2.44 ± 1.33	0.487	0.784 ^‡^
Afternoon	2.27 ± 0.90		
Night	2.30 ± 0.73		
Physical workload			
Somewhat/Moderately load	2.26 ± 0.70	0.238	0.812
Very/Extremely load	2.36 ± 1.04		
Mental stress level			
Somewhat/Moderately stressful	2.53 ± 0.92	1.132	0.292
Very/Extremely stressful	2.20 ± 0.91		
Confidence level			
Somewhat/Moderately confident	2.30 ± 1.03	0.806	0.820
Very/Extremely confident	2.35 ± 0.81		
Attended ACLS training course			
Yes	2.54 ± 0.88	0.908	0.364
No	2.22 ± 0.93		
Attended PALS training course			
Yes	2.80 ± 1.30	1.068	0.285
No	2.26 ± 0.85		

The § P value was calculated using the Mann‒Whitney Z test.

‡ P value was calculated using the Kruskal‒Wallis H test.

** Significant at the p < 0.05 level.

### The relationship between the three levels of SA

Spearman’s correlation analysis was conducted to examine the relationships between the three levels of SA. As shown in Table 5, a significant negative correlation was observed between the perception of patient cues and comprehension (r = −0.361, p < 0.05). No other significant correlations were identified between the remaining SA levels.

**Table 5:** Correlations between different SA levels (n = 40).

Variables	Environmental perception	Patient cues perception	Comprehension	Projection
Environmental perception	1			
Patient cues perception	−0.150	1		
Comprehension	0.194	−0.361*	1	
Projection	−0.126	−0.053	0.234	1

* Correlation is significant at the 0.05 level (2-tailed).

## Discussion

This study aimed to examine the SA of nurses from two hospitals during patient triage to determine the priority of being seen by a physician. All participants were ED nurses, and the study approach addressed several methodological challenges commonly associated with SA assessment. First, SA was assessed in an authentic, real-world context rather than a simulated or semi-real environment. While simulation-based assessments are often recommended to enhance patient safety [25], it can be challenging to accurately replicate the complexity and unpredictability of a real working environment [26]. For instance, Sinz et al. [26] questioned the reliability of simulation-based performance assessments under complex, dynamic conditions. By conducting the assessment in a genuine ED setting, this study provides insights grounded in the actual workflow and challenges faced by nurses.

Second, the study utilized the SAGAT, an objective and flexible method of evaluating SA. Unlike other methods, SAGAT does not impose rigid constraints on when and where freezes occur during assessments. As noted by Endsley [27], this flexibility reduces the likelihood of participants relying on memory recall to provide correct answers, thereby enhancing the validity of the assessment. Additionally, SAGAT is highly adaptable, allowing it to be tailored to various situations and effectively used in real-life clinical contexts [28].

A particularly concerning finding in this study is that the percentage of participants achieving full SA was as low as 10%. This indicates that only a small proportion of participants were able to correctly perceive situational items (both clinical and environmental), interpret the information, and accurately predict the outcomes of the situation. This result is significantly lower than what has been reported in previous literature. For example, Parush et al. [29] employed SAGAT in simulated emergency scenarios and found that 64.6% of healthcare providers demonstrated full SA. Poor SA is widely recognized as a key factor contributing to errors in clinical practice [30]. The low SA scores observed in the current study may be attributed to the frequent workflow interruptions that were noted during the data collection period. Such interruptions, which are common in ED settings, have been shown to impede cognitive processes and diminish SA among nurses [15]. This underscores the need for strategies aimed at mitigating workflow disruptions to enhance SA in ED environments.

The findings of the current study revealed that nurses’ perceptions of patients’ cues were significantly reduced by workload (p = 0.048) and mental stress (p = 0.025). The relationship between workload and perception levels is well-documented in both theoretical and empirical studies [31,32]. Workload has been shown to interfere with an individual’s ability to adequately perceive critical information, particularly in high-stakes environments such as the ED [31]. Similarly, stress can impair perception by causing individuals to miss or fail to process events occurring directly in front of them, a phenomenon known as inattentional blindness [31]. Inattentional blindness occurs when individuals are unable to consciously perceive cues within their visual field due to cognitive overload or distraction [33,34].

Failure to perceive patient cues has been strongly associated with negative outcomes for patients, as it can lead to delayed or incorrect clinical decision-making [35]. However, the results of this study also indicated that workload and stress did not significantly influence comprehension or projection levels. This may be because these mental constructs depend more heavily on information processing and cognitive synthesis rather than on initial sensory perception [36]. These findings emphasize the need for strategies to mitigate workload and stress in EDs to ensure accurate perception and, consequently, better patient care outcomes.

The results of the current study showed that the perception of patient cues was significantly higher among participants who played video games more frequently than their counterparts (p = 0.014). The potential of video games to enhance perceptual abilities has been explored in both cognitive science and healthcare research [19,20]. Specifically, Al-Moteri et al. [20] demonstrated that nursing students who engaged in video gaming exhibited significantly improved visual search abilities and clinical observation skills compared to non-gaming students. These findings align with the current study’s results, suggesting that video gaming could positively influence the perceptual aspects of SA in ED nurses. Given the fundamental role of clinical observation in ED settings, incorporating attention training activities—such as those provided by certain types of video games—may offer a novel and practical approach to enhancing nurses’ perceptual skills. This could ultimately improve patient care outcomes by strengthening the ability of ED nurses to recognize critical cues in high-pressure environments.

An intriguing finding in this study is the significant negative correlation observed between the perception of patient cues and comprehension, which highlights a potential cognitive trade-off between these two levels of SA. This finding suggests that as participants focus on perceiving patient cues (Level 1), their ability to synthesize and interpret this information into a meaningful understanding of the patient’s condition (Level 2) may diminish, and vice versa. This inverse relationship may stem from the cognitive demands placed on triage nurses in high-pressure environments, where attention and mental resources are often stretched to their limits. For instance, the urgency to gather patient cues quickly in a busy ED may restrict the cognitive bandwidth available for deeper comprehension of the collected information. Conversely, prioritizing comprehension may detract from attentiveness to immediate environmental cues. This dynamic is consistent with Endsley’s situational awareness theory, which underscores the limitations of human cognitive processing in dynamic and high-stakes environments [27]. Furthermore, research shows that factors such as cognitive overload, stress, and time constraints can hinder nurses’ ability to effectively balance different SA levels [15]. Future research should focus on developing targeted strategies to address this trade-off, including cognitive training designed to enhance multitasking and decision-making capabilities under time-critical conditions. Such interventions have the potential to improve the overall SA of nurses, thereby enhancing patient safety and care outcomes in emergency settings.

Studies conducted in aviation and driving have demonstrated that the majority of errors occur at the perception level [37,38]. Endsley emphasizes the critical role of physical space design, such as optimizing cockpit layouts, to improve perception in aviation [38]. Similarly, these principles can be applied to EDs by incorporating appropriate equipment and redesigning the physical layout to enhance nurses’ perception during the triaging process. While further research is necessary to fully understand the development and application of the SA concept among ED nurses, particularly during triage, the findings of the current study offer valuable insights. These results hold potential for informing future practices, educational initiatives, and research efforts aimed at improving SA and ultimately enhancing patient safety in ED settings.

## Practical implications

The findings of this study underscore the critical need for targeted interventions to enhance SA among triage nurses in EDs. A key recommendation is the implementation of structured SA training programs that simulate the high-pressure scenarios typical in ED settings. Such training could leverage immersive simulations or rapid visual search games, both of which have been shown to improve observational and decision-making skills. Tailoring these programs to address specific challenges such as workload, stress, and environmental distractions could further enhance their effectiveness.

Additionally, minimizing noise levels and reducing overcrowding in triage areas can help to decrease distractions, allowing nurses to better focus on critical tasks. Regular team-based drills and collaborative exercises emphasizing SA and communication could further strengthen shared awareness among healthcare teams. By simulating high-pressure scenarios, these activities promote effective decision-making and teamwork. Evidence supports the effectiveness of these strategies, highlighting the importance of environmental design and team training in improving patient safety and care outcomes within dynamic healthcare environments [15,27,29]. By integrating these practical measures, this study provides actionable insights into emergency nursing practice and supports the development of evidence-based strategies to meet the complex demands of ED environments.

## Limitation

This study has several limitations that should be acknowledged for a balanced interpretation of the findings. One limitation is the potential influence of the observer’s presence on participants’ behavior, commonly referred to as the “Hawthorne effect” [39]. The presence of an observer in the ED may have led participants to consciously or unconsciously adjust their triage behavior to align with perceived expectations. This adaptation could have inflated the observed levels of SA or altered workflow patterns during the freeze probe sessions. To mitigate this effect, the observational process was designed to blend seamlessly into the natural ED workflow. The researcher maintained a non-intrusive presence, and data collection occurred during routine triage activities to minimize disruptions. Another limitation is the cross-sectional nature of the study, which captures SA at a single point in time and may not reflect dynamic changes across shifts or extended periods. Additionally, the study was conducted in two MOH tertiary hospitals in Saudi Arabia, which may limit the generalizability of the findings to other healthcare settings with different resource availability and triage protocols.

Finally, while the freeze probe technique is a valid method for assessing SA, its reliance on timed interruptions poses a potential risk of influencing participants’ task focus. Despite efforts to conduct freezes during non-critical moments to avoid disrupting patient care, the artificial nature of the interruptions may not fully replicate real-world distractions faced by ED nurses. Future research should address these limitations by reducing the observer’s impact, exploring situational awareness in diverse settings, and employing longitudinal designs to provide a more comprehensive understanding of its implications for clinical practice.

## Conclusion

The study showed that inadequate SA is evident among the observed ED nurses in the two participating hospitals. SA remains critical for an effective triaging process in EDs. SA can be compromised by workload and cognitive stress due to distractions and interruptions. To acquire the necessary skills for adequate SA, nurses must be repeatedly exposed to stressful, attention-focused video games. The study results provide valuable insights into the SA concept among ED nurses and can inform future practice, education, and research. However, larger-scale studies involving diverse populations and healthcare contexts are needed to expand the knowledge gained from this research and enhance its generalizability.
